# A Case of Traumatic Tetanus Successfully Treated by Anæsthetic Agents

**Published:** 1853-11

**Authors:** T. F. Betton

**Affiliations:** Borough of Germantown, Philadelphia county


*A Case of Traumatic Tetanus successfully treated by Anæsthetic agents.* By T. F. Betton, M. D., of the Borough of Germantown, Philadelphia county.

On Thursday, September 22d, 1853, I was called to see a young man of some nineteen or twenty years of age, boarding at Rockville Place, (the residence of Capt. J. D. Miles,) in this borough. He was supposed to be affected with cholera, or yellow fever, and the alarm of the inmates of the house was not inconsiderable. I saw him about 6 o'clock, P. M., and found him laboring under intense pain in the nape of the neck and back of the head, and also in the right hypochondriac region, extending towards the epigastrium. The history of the case given to me was, that he had arrived in the 11 o'clock train from Philadelphia, and walked over to Rockville, a distance of one and a half miles, feeling very well; nothing peculiar was observed about him. He himself informed me that about 3 o'clock, P. M., feeling a desire to evacuate his bowels, on retiring for that purpose he was seized with great pain in the bowels, accompanied by vomiting and purging. These circumstances, with the addition of a violent convulsion, gave rise to the idea of cholera. When I saw him he was strongly convulsed and agitated by the spasms so characteristic of tetanus, (opisthotonos,) which I, at once, supposed the disease to be. On questioning him, however, as to whether he had recently received any sort of wound, he replied in the negative. I then concluded that I was mistaken, but his mind, up to that time, not being very clear, I determined to act on my first impulse, and treat the case as one of tetanus. I gave him, at once, a large dose of Hoffman's anodyne, and tr. valerian with tr. opii, the only remedies at hand. His spine was also freely rubbed with a liniment composed of aq. ammon. fort., ol. succ. rect., spts. terebinth, and tr. opii, in equal parts. A pill of one grain of calomel, and half a grain of opium, was directed to be given hourly. I visited him at 10 P. M., and found the spasms still as strong as ever, but his mind being somewhat improved, he informed me that some three weeks previously, (in this he was incorrect, it was but one week,) he had been hurt by a nail in his foot. On examining his foot, no mark of injury could be discovered, but great tenderness on pressure existed near the ball of the great toe. I made a deep incision in the part affected, without any apparent benefit. Continued the remedies during the night, with the addition of tr. cantharid., and tr. capsici to the liniment.

Friday, 8 o'clock, A. M.—Condition worse. Inability to swallow, difficulty of speech, and, thinking himself dying, he had taken leave of his friends. Spasms increasing in frequency and intensity. Having provided myself with a mixture of three parts of sulphuric ether to one of chloroform, I instantly caused him to inhale it. The result was most gratifying. The spasm ceased as soon as the anæsthetic exerted its influence, and he became calm. In a few moments he was able to swallow some brandy and water. The original treatment was continued, with directions to let him inhale the ether whenever a spasm began to manifest itself. 3 o'clock, P. M.—Progressing favorably, spasms perfectly controlled by the remedies, which were continued. 7½ o'clock, P. M.—My friend Dr. Ashmead, of this borough, saw him with me, and approving of the treatment, it was continued. My friends, Dr. Squire, and Mr. Henry, student of medicine, having kindly consented to watch him during the night, he was left to their care, and I have sincere pleasure in saying that, to their judicious attention, skilful nursing, and management of the anæsthetic, his life is mainly due. A mixture of chloric ether and chloroform was freely administered during the night, (about every fifteen minutes for four hours,) whenever an indication of spasm was present. Beef tea and brandy and water were also given.

Saturday, 8 o'clock, A. M.—Much improved, free from spasm. Seven o'clock, P. M.—Doing well, had a slight spasm at eleven A. M., and one, more slight, at three, P. M., none since.

Sunday.—This day was passed in tranquillity, his appetite is good, and condition in every way favorable.

Monday, 9 o'clock, A. M.—Improving rapidly, and may be fairly considered convalescent. He has been entirely free from spasm for about forty-two hours.

Tuesday.—Has discharged his doctor, and considers himself well.

I now regret that I did not employ the chloroform on Thursday evening, instead of waiting until Friday morning, as he would have been saved some spasms. To the anæsthetic *alone* I

attribute his recovery, and its influence appeared almost miraculous. May it not be equally valuable in hydrophobia?

